# A Tryptophan Metabolite, 8-Hydroxyquinaldic Acid, Exerts Antiproliferative and Anti-Migratory Effects on Colorectal Cancer Cells

**DOI:** 10.3390/molecules25071655

**Published:** 2020-04-03

**Authors:** Katarzyna Walczak, Ewa Langner, Karolina Szalast, Anna Makuch-Kocka, Piotr Pożarowski, Tomasz Plech

**Affiliations:** 1 Department of Pharmacology, Medical University of Lublin, Chodźki 4a, 20-093 Lublin, Poland; ewa.langner@umlub.pl (E.L.); karolina.szalast@umlub.pl (K.S.); anna.makuch@umlub.pl (A.M.-K.); 2 Department of Medical Biology, Institute of Rural Health, Jaczewskiego 2, 20-090 Lublin, Poland; 3 Chair and Department of Clinical Immunology, Medical University of Lublin, Chodźki 4a, 20-093 Lublin, Poland; piotr.pozarowski@umlub.pl

**Keywords:** colorectal cancer, metastasis, Wnt/β-catenin pathway, cell cycle regulators, zebrafish

## Abstract

8-Hydroxyquinaldic acid, the end-metabolite of tryptophan, is well-known metal chelator; however, its role in humans, especially in cancer promotion and progression, has not been fully revealed. Importantly, 8-hydroxyquinaldic acid is the analog of kynurenic acid with evidenced antiproliferative activity towards various cancer cells. In this study, we revealed that 8-hydroxyquinaldic acid inhibited not only proliferation and mitochondrial activity in colon cancer HT-29 and LS-180 cells, but it also decreased DNA synthesis up to 90.9% for HT-29 cells and 76.1% for LS-180 cells. 8-Hydroxyquinaldic acid induced changes in protein expression of cell cycle regulators (CDK4, CDK6, cyclin D1, cyclin E) and CDKs inhibitors (p21 Waf1/Cip1, p27 Kip1), but the effect was dependent on the tested cell line. Moreover, 8-hydroxyquinaldic acid inhibited migration of colon cancer HT-29 and LS-180 cells and increased the expression of β-catenin and E-cadherin. Importantly, antiproliferative and anti-migratory concentrations of 8-hydroxyquinaldic acid were non-toxic in vitro and in vivo. We reported for the first time antiproliferative and anti-migratory activity of 8-hydroxyquinaldic acid against colon cancer HT-29 and LS-180 cells.

## 1. Introduction

Colorectal cancer (CRC) is a growing problem in highly developed countries and the second cause of death among patients diagnosed with cancer in the world [1]. Although CRC develops for about 10–20 years, late diagnosis is one of the causes of poor treatment efficacy. Nowadays, the new methods of CRC treatment are focused on three issues: chemoprevention, effective prevention of metastases, and CRC recurrence [2]. 8-Hydroxyquinaldic acid was shown to derive from tryptophan metabolism [3]. It is the end-metabolite of tryptophan [4], a product of dehydroxylation of xanthurenic acid [5]. 8-Hydroxyquinaldic acid is well-known metal chelator [3,6]; however, its role in humans has not been fully revealed. Interestingly, 8-hydroxyquinaldic acid is the analog of kynurenic acid which antiproliferative activity towards various cancer cells was evidenced previously [7,8,9,10]. Kynurenic acid, affecting MAPK and PI3K/Akt signaling pathways and the expression of cell cycle regulator p21 Waf1/Cip1, inhibits proliferation and migration of various cancer cells including colon cancer cells [7,9,10,11]. Structural similarity to kynurenic acid may suggest the potential anti-cancer activity of 8-hydroxyquinaldic acid; however, its biological activity, especially towards cancer cells, has not been studied so far. Interestingly, studies conducted in the 1960s and 1970s in rats and patients with bladder cancer showed that various metabolites of the kynurenine pathway, including 8-hydroxyquinaldic acid, are carcinogenic [12,13]. However, further studies revealed that only a small percentage of patients with bladder cancer had abnormalities in tryptophan metabolism and increased levels of metabolites in the urine [14,15]. Taking into consideration previous ambiguous results and structural similarity to kynurenic acid, we decided to investigate the toxicity of 8-hydroxyquinaldic acid and its effect on colon cancer cell proliferation, the cell cycle, and migration in vitro. Additionally, the molecular mechanism of the biological activity of 8-hydroxyquinaldic acid in colon cancer HT-29 and LS-180 cells was determined.

## 2. Results

**Figure 1:**
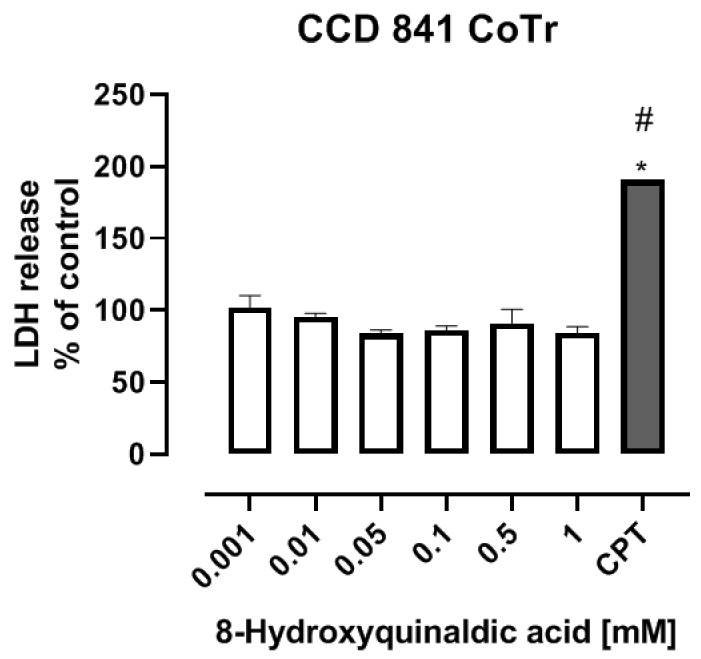
The effect of 8-hydroxyquinaldic acid on viability of human colon epithelial CCD 841 CoTr cells. CCD 841 CoTr cells were exposed to culture medium supplemented with 2% fetal bovine serum (FBS) (control) or serial dilutions of 8-hydroxyquinaldic acid (0.001; 0.01; 0.05; 0.1; 0.5; 1 mM) in culture medium supplemented with 2% FBS for 24 h. The toxicity of 8-hydroxyquinaldic acid was assessed by the LDH method. Data represent a mean value (% of control) ± SEM from six independent experiments (the mean value of control = 100%). **#**: positive control, 25 μMcamptothecin(CPT)—the cytotoxic effect of CPT on CCD 841 CoTr cells was previously reported by Czerwonka et al. (2020) [16]. Values significant (*) in comparison to the control at *p* < 0.05 (one-way ANOVA with post hoc Tukey test).

**Figure 2:**
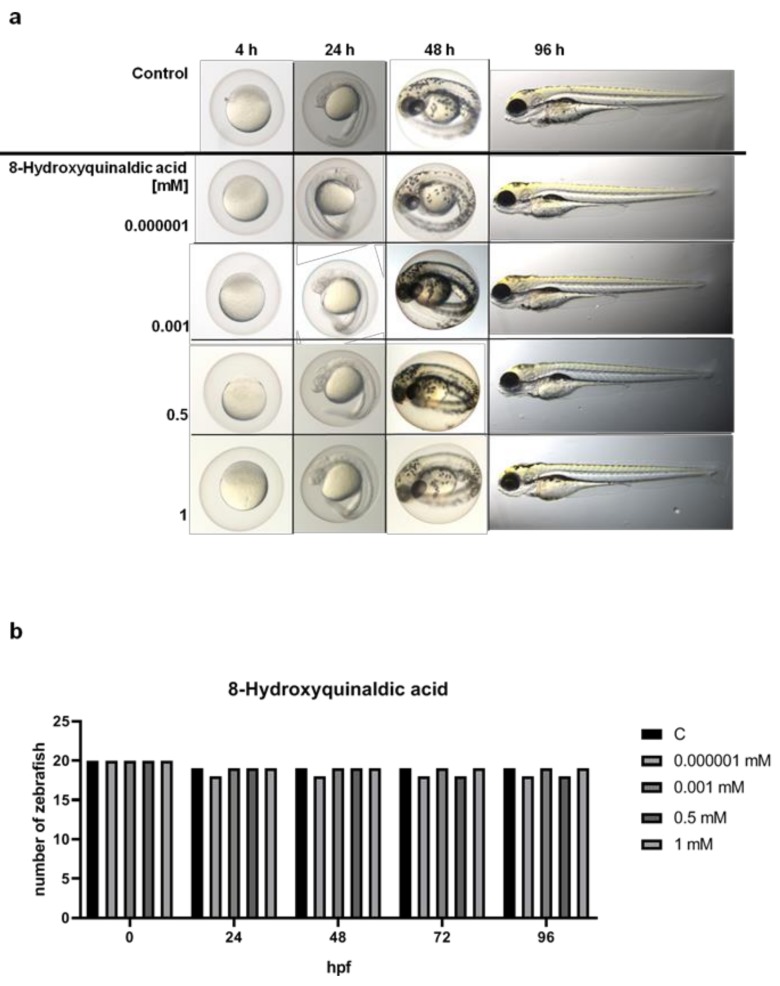
The effect of 8-hydroxyquinaldic acid on development (**a**) and viability (**b**) of zebrafish larvae. Fertilized zebrafish (AB line) eggs (*n* = 20) were exposed to E3 medium (control, C) or serial dilutions of 8-hydroxyquinaldic acid (0.000001; 0.001; 0.5, 1 mM) in E3 medium for 96 h. The medium was changed every 24 h. The microscopic observations were made in selected time points, i.e., 4, 24, 48, 72, 96 h.

To study the toxicity of 8-hydroxyquinaldic acid, we evaluated the cytotoxic effect of the tested compound on colon epithelial cells in vitro and its activity on development of zebrafish embryos and larvae. CCD 841 CoTr cells were exposed to series of substance concentrations ranged from 0.001 to 1 mM. 8-Hydroxyquinaldic acid did not induce cytotoxicity in colon epithelial cells in vitro after 24 h incubation (Figure 1), in contrast to camptothecin(CPT), a positive control, reported previously by Czerwonka et al. [16].Camptothecin increased lactate dehydrogenase (LDH) release up to 190.7% ± 3.959% (% of control ± SEM) [16]. Importantly, we did not observe any significant disorders in embryonic development of zebrafish larvae treated with 8-hydroxyquinaldic acid (0.000001–1 mM) (Figure 2). The tested concentrations were non-toxic for zebrafish and did not affect the hatching of zebrafish larvae. Importantly, a toxic effect of camptothecin, a positive control in in vitro LDH assays, on embryonic development of zebrafish was previously reported by Li et al. [17].

**Figure 3:**
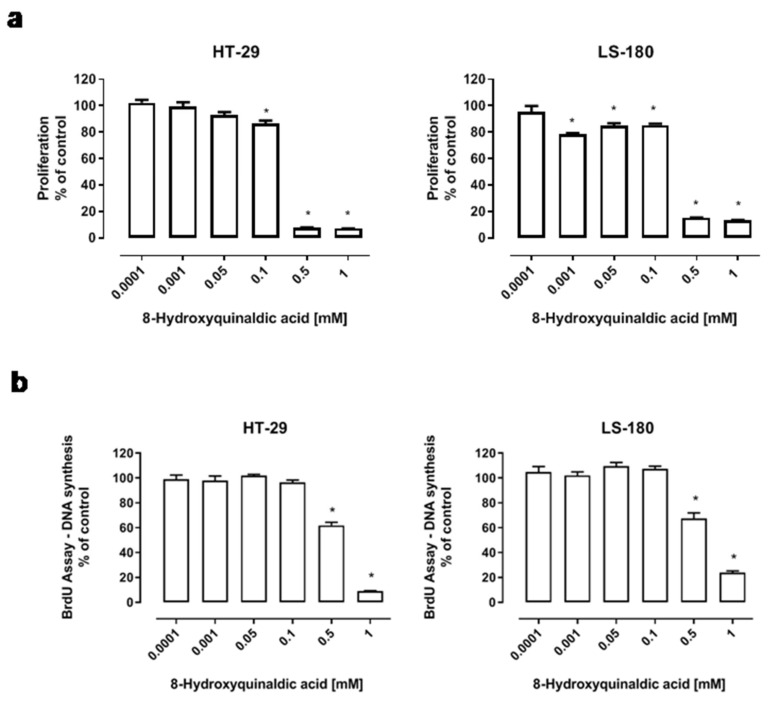
The effect of 8-hydroxyquinaldic acid on proliferation (**a**) and DNA synthesis (**b**) of human colon adenocarcinoma cells. HT-29 and LS-180 cells were exposed to culture medium (control) or serial dilutions of 8-hydroxyquinaldic acid (0.0001; 0.001; 0.05; 0.1; 0.5; 1 mM) in culture medium. The effect of tested compound on proliferation and metabolic activity of HT-29 and LS-180 cells was assessed by MTT assay after 96 h of incubation (**a**). The effect of 8-hydroxyquinaldic acid on DNA synthesis of HT-29 and LS-180 cells was assessed by BrdUassay after 48 h of incubation (**b**). Mean percentage values (% of control) ± SEM of six independent experiments (the mean value of control = 100%) are presented. Values were reported as statistically significant in comparison to the control at * *p*< 0.05 (one-way ANOVA with Tukey post hoc test).

To determine the biological effect of 8-hydroxyquinaldic acid on colon cancer cells, several in vitro experiments on human HT-29 and LS-180 colon cancer cells were performed. The effect of 8-hydroxyquinaldic acid on colon cancer cell viability and proliferation was assessed by MTT and BrdU assays, which measure the activity of mitochondrial metabolism and DNA synthesis, respectively. HT-29 and LS-180 cells were exposed either to fresh medium or to 8-hydroxyquinaldic acid (0.0001–1 mM) for 48 h (BrdU assay) or 96 h (MTT assay). 8-Hydroxyquinaldic acid inhibited the viability and mitochondrial metabolism of HT-29 (IC50 = 0.175 mM) and LS-180 (IC50 = 0.349 mM) cells (Figure 3a). Importantly, the inhibitory effect in LS-180 cells was observed in a wide range of concentrations (0.001–1 mM). However, only the highest doses of 8-hydroxyquinaldic acid (0.5–1 mM) decreased DNA synthesis in HT-29 and LS-180 cells up to 90.9% (IC50 = 0.774 mM) and 76.1% (IC50 = 0.567 mM), respectively (Figure 3b).

**Figure 4:**
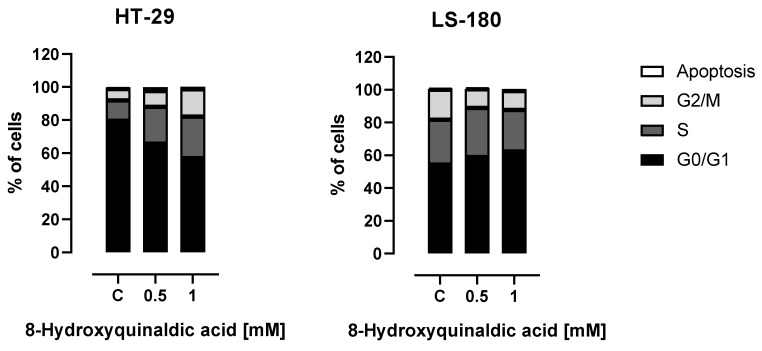
The effect of 8-hydroxyquinaldic acid on the cell cycle in human colon adenocarcinoma cells. HT-29 and LS-180 cells were exposed to culture medium (control, C) or selected dilutions of 8-hydroxyquinaldic acid (0.5; 1 mM) in culture medium for 24 h. The cell cycle progression was assessed in a flow cytometer using the propidium iodide method described previously in [18]. The experiment was run in triplicate. Cell separation to G0/G1, S, and G2/M phases and the apoptotic groupwas presented as a percentage of total number of cells.

To shed some light on the molecular mechanism of antiproliferative action of 8-hydroxyquinaldic acid towards colon cancer cells, the influence of the tested compound on the cell cycles of HT-29 and LS-180 cells was studied. Interestingly, the cell cycle analysis revealed different effects of 8-hydroxyquinaldic acid on the cell cycle progression in HT-29 and LS-180 cells. A significant decrease in the number of cells in the G0/G1 phase followed by the increase of cells in S and G2/M phases was observed in HT-29 cells exposed to 8-hydroxyquinaldic acid. However, 8-hydroxyquinaldic acid induced the increase in G0/G1 phase in LS-180 cells with simultaneous diminishing the number of cells in G2/M phase (Figure 4).

**Figure 5:**
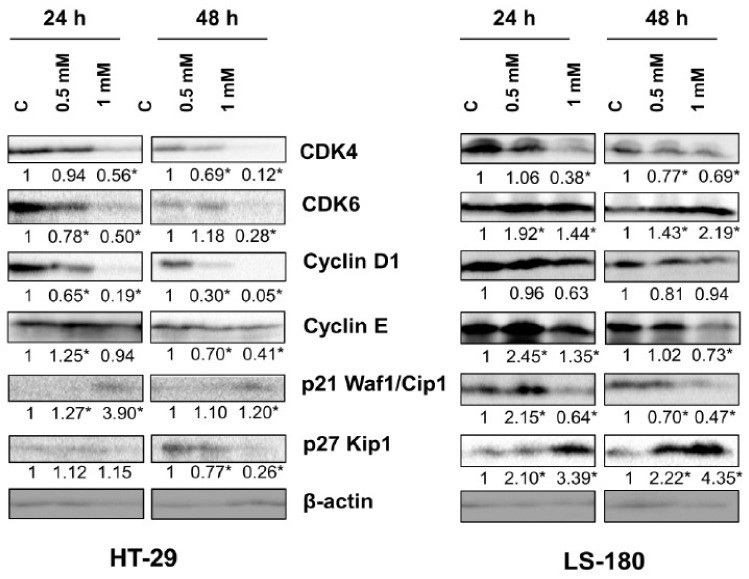
The effect of 8-hydroxyquinaldic acid on protein expression of selected cell cycle regulators in human colon adenocarcinoma cells. Western blot analysis of selected cell cycle regulators was performed after treatment of HT-29 and LS-180 cells with 8-hydroxyquinaldic acid (0.5, 1 mM) for 24 and 48 h (C control, not treated). Western blots shown in the figure were selected as the most representative of the series of repetitions *n* ≥ 3. The data were normalized to β-actin and calculated fold changes in protein expression marked (fold changes ≥ 0.20 were considered as significant [*]).

To study the molecular basis of these interactions, the effect of 8-hydroxyquinaldic acid on the expression of cell cycle regulators in HT-29 and LS-180 cells was analyzed by means of Western blot. 8-Hydroxyquinaldic acid decreased the expression of cyclin D1 and CDK4 in HT-29 and LS-180 after 24 and 48 h (Figure 5). The similar effect of 8-hydroxyquinaldic acid on cyclin E expression was observed in both HT-29 and LS-180 cells; however, the effect was stronger in LS-180 cells. The tested compound increased its expression after 24 h of incubation, but after 48 h expression of cyclin E was decreased.However, the effect on protein expression of CDK6, p21 Waf1/Cip1, and p27 Kip1 was different for HT-29 and LS-180 cells. Incubation for 48 h with 8-hydroxyquinaldic acid resulted in inhibition of CDK6 in HT-29 cells, whereas increased expression of CDK6 was observed in LS-180 cells treated with the tested compound (Figure 5). Interestingly, 8-hydroxyquinaldic acid (1 mM) increased expression of p21 Waf1/Cip1 after 24 and 48 h in HT-29 cells, but it decreased expression of this protein in LS-180 cells. Additionally, 8-hydroxyquinaldic acid increased protein expression of p27 Kip1 in a concentration-dependent manner in LS-180 cells but not in HT-29 cells (Figure 5).

**Figure 6:**
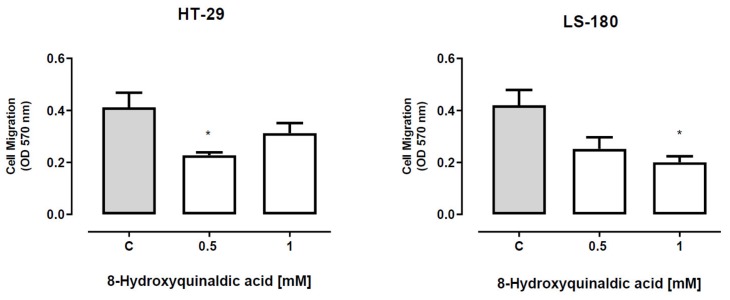
The effect of 8-hydroxyquinaldic acid on migration of human colon adenocarcinoma cells. The migration of HT-29 and LS-180 cells through a monolayer of human umbilical vein endothelial HUVEC cells was assessed byMillipore′s QCM tumor cell transendothelial cell migration assay. Data represent the OD value (570 nm) ± SEM of three independent experiments. The absorbance was proportional to the migration of tumor cells.Results were designated as statistically significant in comparison to the control (C) when * *p*< 0.05 (unpaired Student’s t-test).

To verify whether 8-hydroxyquinaldic acid has any effect on the migration of colon cancer cells, the ability of HT-29 and LS-180 cells exposed to tested compound to invade the endothelial cell monolayer was analyzed by means of QCM™ tumor cell transendothelial migration assay. 8-Hydroxyquinaldic acid in the concentration of 0.5 mM, but not 1 mM, inhibited migration of HT-29 cells (Figure 6). Statistically significant inhibitory effect was also observed in LS-180 cells exposed to 8-hydroxyquinaldic acid (1 mM) (Figure 6).

**Figure 7:**
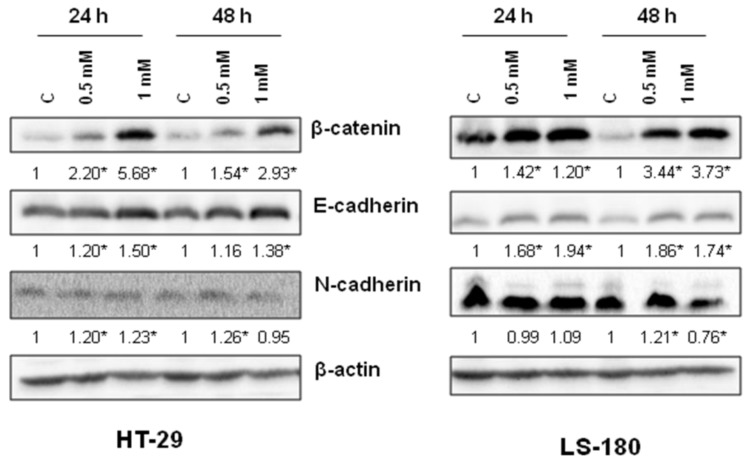
The effect of 8-hydroxyquinaldic acid on expression of β-catenin, E- and N-cadherin in human colon adenocarcinoma cells. Western blot analysis of expression of β-catenin and E- and N-cadherin was performed after 24 and 48 h treatment of HT-29 and LS-180 cells with 8-hydroxyquinaldic acid (0.5, 1 mM). Western blots presented above were selected as the most representative of the series of repetitions *n* ≥ 3. The data were normalized relative to β-actin and calculated fold changes in protein expression marked (fold changes ≥ 0.20 were considered as significant [*]).

**Figure 8:**
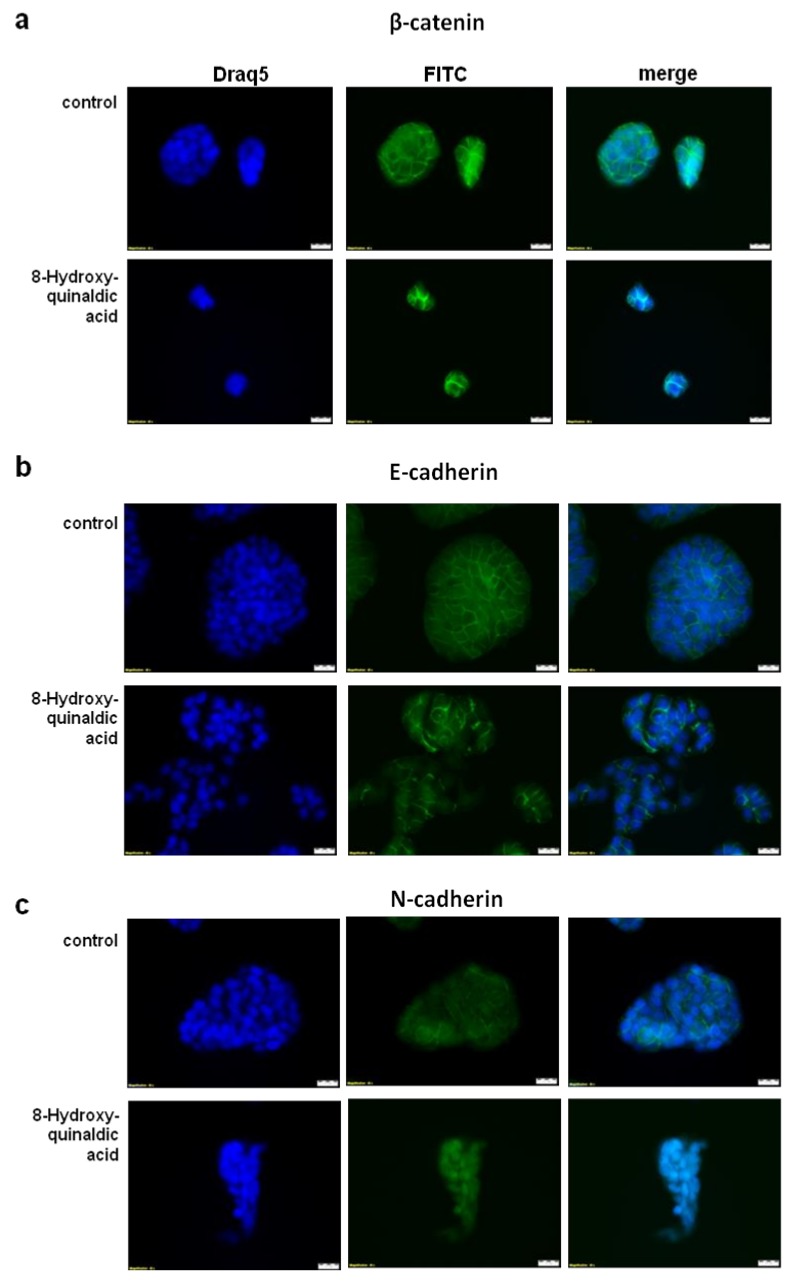
The effect of 8-hydroxyquinaldic acid on cellular localization of β-catenin (**a**), E- (**b**) and N-cadherin (**c**) in HT-29 cells. Immunofluorescent staining for β-catenin and E- and N-cadherin in HT-29 cells exposed to 8-hydroxyquinaldic acid (1 mM) for 48 h (control; not treated). Cell nuclei were labeled with cell permeable fluorescent DNA dye DraQ5. Magnification was 40×.

To examine the molecular basis of anti-migratory activity of 8-hydroxyquinaldic acid in HT-29 and LS-180 cells, the expression of proteins involved in this process, including β-catenin and E- and N-cadherin, was studied. 8-Hydroxyquinaldic acid in a dose-dependent manner enhanced expression of β-catenin and E-cadherin after 24 and 48 h (Figure 7). Only slight changes were observed in the expression of N-cadherin in HT-29 and LS-180 cells (Figure 7). Importantly, although the protein expression profile did not explain the differences in migration of colon cancer HT-29 and LS-180 cells (Figure 7), the immunofluorescent staining revealed nuclear translocation of N-cadherin in HT-29 cells exposed to 8-hydroxyquinaldic acid for 48 h (Figure 8).

## 3. Discussion

Although 8-hydroxyquinaldic acid, the end-metabolite of tryptophan degradation, was discovered in the last century [4,5,12], its biological activity and potential role in humans have not been fully revealed. Its physicochemical properties for metal chelation are currently used in some chemical studies [6,19,20], however, for the first time we focused on the toxicity and potential anti-cancer properties of 8-hydroxyquinaldic acid towards colon cancer cells.

According to the structural similarities with kynurenic acid and the biological activities of 8-hydroxyquinaldic acid, it may be considered as a new agent involved in the chemoprevention or therapy of colon cancer. 8-Hydroxyquinaldic acid inhibited colon cancer cell proliferation and mitochondrial metabolism in a dose-dependent manner. The inhibitory potential of the tested compound on mitochondrial metabolism assessed by the MTT method was observed atthe concentration of 0.1 mM in HT-29 cells (IC50 = 0.175 mM) and at0.001 mM in LS-180 cells (IC50 = 0.349 mM)(Figure 3a). Importantly, 8-hydroxyquinaldic acid also inhibited DNA synthesis in both colon cancer cell lines (HT-29, IC50 = 0.774 mM; LS-180, IC50 = 0.567 mM) (Figure 3b). Surprisingly, these results indicate a stronger antiproliferative effect in HT-29 colon cancer cells of 8-hydroxyquinaldic acid in comparison to well-studied kynurenic acid (HT-29, MTT IC50 = 0.9 mM [7]; BrdU IC50 = 4.4 mM [9]). It may be suggested that slight changes in the chemical structure have a significant impact on the biological properties of these compounds.

High concentrations of 8-hydroxyquinaldic may be a potential problem for the future use of this compound in colon cancer chemoprevention or treatment of colon cancer. However, as the end-metabolite of tryptophan degradation, it is probably naturally produced in the human body. Unfortunately, there is no study indicating its physiological concentration in the various tissues and body fluids. Interestingly, 8-hydroxyquinaldic acid was found in high concentrations (0.5–5 mM) in the regurgitate of Spodoptera larvae [3]. Therefore, we decided to study the toxicity profile of 8-hydroxyquinaldic acid in vitro and in vivo. Importantly, 8-hydroxyquinaldic acid was non-toxic for colon epithelial CCD 841 CoTr cells in vitro, even atthe concentration of 1 mM (Figure 1), and did not significantly affect the mortality of zebrafish embryos and larvae (Figure 2). Moreover, 8-hydroxyquinaldic acid did not induce any severe defects in zebrafish embryogenesis or organogenesis (Figure 2). In contrast, camptothecin, reported as a positive control of cytotoxicity in CCD 841 CoTr cells [16] (Figure 1), induced p53-mediated early apoptosis in zebrafish embryos [17].

Due to the lack of toxicity to normal colon epithelial cells and antiproliferative effects on colorectal cancer cells, we studied the effect of 8-hydroxyquinaldic acid on cell cycle progression and expression of cell cycle regulators. 8-Hydroxyquinaldic acid inhibited the cell cycle in HT-29 and LS-180 cells; however, the observed effects were different for these cell lines, which may suggest that the mechanism of action is different (Figure 4). 8-Hydroxyquinaldic acid induced a significant decrease in the total number of cells in the G0/G1 phase followed by the increase of cells in S and G2/M phases in HT-29 cells, whereas in LS-180 cells it induced the increase in G0/G1 phase with a simultaneous decrease in the number of cells in G2/M phase (Figure 4). The analysis of the expression of cell cycle regulators revealed that 8-hydroxyquinaldic acid inhibited the expression of cyclin D1 and CDK4 in both colon cancer cell lines. However, 8-hydroxyquinaldic acid inhibited CDK6 expression in HT-29 cells, whereas it induced expression of this kinase in LS-180 cells (Figure 5). It cannot be excluded that 8-hydroxyquinaldic acid induced cell cycle arrest in HT-29 and LS-180 cells, not only by the interaction with cyclins and CDKs, but also via upregulation of p21 Waf1/Cip1 or p27 Kip1 proteins. These CDK inhibitors play a crucial role in elimination of damaged cells and cancer prevention, inhibiting the CDK activity and preventing cycle progression. p21 Waf1/Cip1 or p27 Kip1 proteins play important roles in apoptosis, cell cycle arrest, DNA repair, and senescence [21]. Moreover, the differences between two tested colon cancer cell lines should be underlined and may partially explain various biological effects of 8-hydroxyquinaldic acid in HT-29 and LS-180 cells. The HT-29 cell line was derived from well-differentiated primary colorectal adenocarcinoma tumors, whereas the LS-180 cell line represents Dukes′ type B colorectal adenocarcinoma, a more advanced stage of the disease. Importantly, it also has a direct impact on genetic differences between tested cell lines. HT-29 cells possess a mutated form of the tumor suppressor p53 gene, involved in genome stability and integrity [22]. In p53-mutated cells, p21 Waf1/Cip1 plays a crucial role in the induction of cell cycle arrest and apoptosis. Additionally, overexpression of p21 Waf1/Cip1 in p53-mutated cells more frequently results in cell cycle stops duringthe S phase than in the G0/G1 phase in comparison to cells with wild type of p53 gene [23,24]. However, the effect of 8-hydroxyquinaldic acid on other signaling pathways and receptors cannot be excluded. In contrast to the well-known receptor mechanism of action of the analog, kynurenic acid, the biological interactions of 8-hydroxyquinaldic acid are relatively poorly studied.

In the present study we also investigated the anti-migratory activity of 8-hydroxyquinaldic acid in HT-29 and LS-180 cells. Interestingly, the tested compound inhibited migration of HT-29 cells though the endothelial monolayer atthe concentration of 0.5 mM but not athigher 1 mM concentration (Figure 6). Unexpectedly, the expression of proteins involved in invasion and migration of cancer cells revealed that 8-hydroxyquinaldic acid enhanced β-catenin and E-cadherin expression in both colon cancer cell lines (Figure 7). β-Catenin is the key downstream effector in the Wnt/β-catenin pathway, activation of which leads to the accumulation of β-catenin in the nucleus [25]. Previous studies report involvement of the Wnt/β-catenin pathway in early embryonic development and tumorigenesis [26]. Interestingly, elements of the Wnt/β-catenin pathway are mutated in approximately 90% of CRCs [25] and high levels of nuclear β-catenin have been correlated with a poor prognosis in CRC patients [27]. However, immunofluorescent staining of β-catenin did not confirm its nuclear translocation in HT-29 cells. 8-Hydroxyquinaldic acid also enhanced expression of E-cadherin in HT-29 and LS-180 cells, which plays an important role in tissue development, differentiation, and maintenance, and is also considered as a tumor suppressor [28]. It cannot be excluded that 8-hydroxyquinaldic acid-mediated overexpression of β-catenin is compensated in HT-29 cells by overexpression of E-cadherin, preventing nuclear translocation of β-catenin [28,29]. Importantly, we did not observe any significant changes in N-cadherin expression in HT-29 and LS-180 cells, because overexpression of N-cadherin may promote increased cancer cell motility, migration, and invasion [30]. However, loss of anti-migratory potential of 1 mM 8-hydroxyquinaldic acid in HT-29 cells might be induced by nuclear translocation of this protein indicated by immunofluorescent staining (Figure 8). It should be underlined that several various signaling pathways (i.e., MAPK, PI3K/Akt signaling pathways), transcription factors (i.e.,TWIST1, Snail, Slug, ZEB1/2), and cytokines (i.e., VEGF, TGFβ) may be involved in the migration and invasiveness of cancer cells [22]. Thus, it cannot be excluded that anti-migratory activity of 8-hydroxyquialdic acid towards colon cancer HT-29 and LS-180 cells may not only be dependent on cadherin and β-catenin expression, but affect other elements involved in this process.

In this study we confirmed the antiproliferative and anti-migratory potential of 8-hydroxyquinaldic acid on colon cancer HT-29 and LS-180 cells. Importantly, in vitro and in vivo experiments confirmed that even high concentrations of this compound were not toxic for normal colon cells (CCD 841 CoTr) and did not induce significant development disorders in zebrafish larvae. Previous studies conducted onSpodoptera larvae revealed that tryptophan content in the diet had a significant impact on the amount of 8-hydroxyquinaldic acid in the regurgitate [3]. Importantly, it was suggested that 8-hydroxyquinaldic acid was produced not by intestinal bacteria but directly by Spodoptera larvae [3]. Thus, future studies should be focused on the potential biological role of 8-hydroxyquinaldic acid in physiological processes in humans and endogenous production of this compound in human tissues and physiological fluids. Our results suggested that biological activity of 8-hydroxyquinaldic acid is not limited to metal chelation but it may directly affect various signaling pathways and cell cycle regulators. It is possible that 8-hydroxyquinaldic acid may have an impact on some physiological processes in the human body, especially in the colon, and in higher concentrations may also affect carcinogenesis and cancer progression. The other issue which should be deeply studied is whether 8-hydroxyquinaldic acid is absorbed from the gastrointestinal tract or if its biological impact is only limited to the local activity on the mucosa and intestinal epithelium. However, taking into consideration the antiproliferative and anti-migratory potential of 8-hydroxyquinaldic acid on colon cancer HT-29 and LS-180 cells, the use of this substance in the prevention of colorectal cancer should be considered.

## 4. Materials and Methods

### 4.1. Drugs

8-Hydroxyquinaldic acid was obtained from Sigma–Aldrich (St. Louis, MO, USA). 8-Hydroxyquinaldic acid was dissolved in 1 M NaOH and then diluted in phosphate buffered saline (PBS). No significant effects of solvents on the morphology and proliferation of normal colon epithelial and colon cancer cells were observed.

### 4.2. Zebrafish Experiment

Embryonic exposures to 8-hydroxyquinaldic acid began ~1 h postfertilization (hpf). Only fertilized Zebrafish eggs (AB line) without any malformation or impurities were selected to the experiment. Twenty embryos were assigned randomly to the control group (E3 medium) or experimental groups in which embryos were exposed to 8-hydroxyqiunaldic acid in E3 medium inthe range of concentrations (0.000001; 0.001; 0.1; 0.5; 1 mM). The embryos were kept in 24-well plates (1 embryo per well) at 28.5 °C with day/nightcycles of 14/10 h. The medium and test solutions were changed every 24 h. The microscopic observations were performed at 4, 24, 48, and 96 hpf. Lethal and sublethal endpoints were assessed to determine the toxicity of 8-hydroxyquinaldic acid as described by Lammer et al. [31]. The experiments were repeated twice. Zebrafish images were captured with a ZEISS SteREO Discovery.V8 microscope and Zen 2.3 lite software (Carl Zeiss Microscopy GmbH, Germany).

### 4.3. Cell Culture

A continuous cell line of human colon epithelium CCD841 CoTr cells were obtained from American Type Culture Collection (ATCC, Manassas, VA, USA). Colon adenocarcinoma HT-29 cells and LS-180 cells were obtained from European Collection of Cell Cultures (ECACC, Salisbury, UK). Colon cancer HT-29 and LS-180 cells were cultured using the 1:1 mixture of Dulbecco’s modified Eagle’s medium (DMEM) and nutrient mixture Ham F-12 supplemented with 10% fetal bovine serum (FBS), 100 U/mL of penicillin, and 100 µg/mL of streptomycin.Human umbilical vein endothelial cells (HUVEC), obtained from Sigma–Aldrich, were cultured in Endothelial Cell Growth Medium. HT-29, LS-180 and HUVEC cells were cultured in a monolayer at 80% confluency in a humidified atmosphere of 5% CO_2_ at 37 °C. CCD 841 CoTr cells were cultured in DMEM supplemented with 10% FBS, 100 U/mL of penicillin, and 100 µg/mL of streptomycin and were maintained in a humidified atmosphere of 95% air and 5% CO_2_ at 33 °C. All reagents were obtained from Sigma–Aldrich.

### 4.4. LDH Method

The in vitro toxicology assay kit, lactic dehydrogenase (LDH) based (Sigma–Aldrich) was used to determine the cytotoxicity of 8-hydroxyquinaldic acid. The assay determining the membrane integrity is based on the reduction of NAD by released cytoplasmic LDH. NADH isutilized in the stoichiometric conversion of a tetrazolium dye. CCD 841 CoTr cells were plated in 96-well plates at the density of 3 × 10^4^ cells/mL. Twenty four hours later, the culture medium was removed and the cells were exposed to fresh medium supplemented with 2% FBS (control) or dilutions of 8-hydroxyquinaldic acid (0.001; 0.01; 0.05; 0.1; 0.5; 1 mM) and incubated for 24 h at standard conditions. The activity of released LDH in supernatants was measured according to the manufacturer’s procedure. The product was quantified spectrophotometrically at 490 nm using a microplate reader (Epoch, BioTek Instruments, Inc., Winooski, VT, USA) equipped with Gen5 software (v. 2.01, BioTek Instruments, Inc.).

### 4.5. MTT Assay

The suspension of HT-29 and LS-180 cells was prepared at a density of 3 × 10^4^ cells/mL and then transferred to 96-well cell culture plates (NUNC, Roskilde, Danmark). Twenty four hours later, the culture medium was removed and the cells were exposed to fresh medium containing 10% FBS (control) or dilutions of 8-hydroxyquinaldic acid (0.0001; 0.001; 0.05; 0.1; 0.5; 1 mM) and incubated for 96 h in standard conditions. To assess cell proliferation and the activity of mitochondrial metabolism, a MTT test based on the cleavage of the yellow tetrazolium salt (3-(4,5-dimethylthiazol-2-yl)-2,5-diphenyltetrazolium bromide,(MTT)) to purple formazan crystals by metabolically active cells was used. After 96 h of treatment, cells were incubated with MTT (5 mg/mL) for 3 h at 37 °C and then with SDS buffer (10% SDS in 0.01 N HCl). The next day, the absorbance was measured at 570 nm using a microplate reader (Epoch, BioTek Instruments, Inc., USA) equipped with Gen5 software (v. 2.01, BioTek Instruments, Inc.).

### 4.6. BrdUAssay

The suspension of HT-29 and LS-180 cells was prepared at a density of 5 × 10^4^ cells/mL and then transferred to 96-well cell culture plates (NUNC). The next day, the culture medium was removed, and the cells were exposed to fresh medium containing 10% FBS (control) or dilutions of 8-hydroxyquinaldic acid (0.0001; 0.001; 0.05; 0.1; 0.5; 1 mM). Proliferation and DNA synthesis was assessed after 48 h by measurement of 5′-bromo-2′-deoxy-uridine (BrdU) incorporation during DNA synthesis, according to the manufacturer’s protocol (Cell Proliferation ELISA BrdU, Roche Diagnostics GmbH, Penzberg, Germany).

### 4.7. Cell Cycle Analysis

Colon cancer HT-29 and LS-180 cells were exposed to culture medium containing 10% FBS (control) or selected dilutions of 8-hydroxyquinaldic acid (0.5 or 1 mM) in culture medium for 24 h in standard conditions. The cell cycle progression was assessed in a FACS Calibur flow cytometer (Becton Dickinson, Franklin Lakes, NJ, USA) using the propidium iodide method described previously in [18].

### 4.8. Western Blot

HT-29 and LS-180 cells were lysed in radioimmunoprecipitation assay (RIPA) buffer (2% NP40 (Tergitol), 1 mM EGTA, 1 mM EDTA, 1 mM Na3VO4, 0.5% sodium deoxycholate, 0.1% SDS, 20 mM NaF, 0.5 mM DTT, 1 mM PMSF, protease inhibitor mixture in PBS, pH 7.4) and centrifuged at 14,000× *g* for 10 min.Protein content in supernatants was determined by BCA protein assay kit (Pierce Biotechnology, Rockford, USA). Supernatants were diluted in sample buffer (30% glycerol, 10% SDS, 0.5 M Tris–HCl, pH 6.8, 0.012% bromophenol blue, 5% β-mercaptoethanol), and boiled for 5 min. For Western blotting, equal amounts of proteins were electrophoresed on 7–14% SDS-PAGE gels and transferred to a PVDF membrane. After blocking for 1 h at room temperature with 5% non-fat dry milk in tris-buffered saline–0.1% Tween 20 (TBS-T), membranes were probed at 4 °C overnight with primary antibodies (1:1000): cyclin D1, cyclin E, cyclin-dependent kinase 4 (CDK4), CDK6, p21 Waf1/Cip1, p27 Kip1, β-catenin, E-cadherin, N-cadherin, β-actin (Cell Signaling Technology, Danvers, USA). The membranes were then washed in TBS-T buffer and incubated with secondary antibody coupled to horseradish peroxidase (1:2000 in 5% non-fat milk in TBS-T; Cell Signaling Technology) for 1 h at room temperature and visualized using enhanced chemiluminescence (Pierce Biotechnology). ImageJ software was used to carry out densitometric analyses.

### 4.9. Migration ECM

Millipore′s QCM tumor cell transendothelial cell migration assay—Colorimetric (Merck Millipore, Burlington, MA, USA) provides a model to analyze the ability of tumor cells to invade the endothelium. HUVEC endothelial cells (1 × 10^5^) in 250 μLof endothelial cell culture medium were added to each insert. The cells were grown for 48 h in standard conditions (to reach ~95% confluence). Then, the endothelial cell culture medium was removed from the cell culture insert and the inserts were transferred to new wells containing 300 μL of serum-free tumor cell growth medium containing 0.5% BSA. HT-29 or LS-180 cells (1 × 10^5^) in 250 μLof tumor cell culture mediumcontaining 0.5% BSA (control) or suspensions of 8-hydroxyquinaldic acid (0.5, 1 mM) were added to the upper well of the inserts. Appropriate negative control inserts without tumor cells (HUVECs only) and with tumor cell culture medium were included. Cells were incubated in standard conditions for 24 h. Then cells from underside of the membrane were stained, extracted, and colorimetrically quantified according to manufacturer’s instruction. The absorbance was proportional to the migration of tumor cells.

### 4.10. Immunofluorescence

HT-29 cells cultured on LabTek Chamber Slides (Nunc, ThermoFisher Scientific, Roskilde, Denmark) were exposed to culture medium (control) or 1 mM 8-hydroxyquinaldic acid for 48 h in standard conditions. Then, cells were fixed with 3.7% paraformaldehyde, permeabilized with 0.2% Triton X-100, and treated with 5% bovine serum albumin (BSA, Sigma–Aldrich). The cells were exposed to primary antibodies against β-catenin and N- and E-cadherin (1:100; Cell Signaling Technology, Danvers, MA, USA) overnight at 4 °C and then were incubated with secondary antibody conjugated with fluorescein isothiocyanate (FITC)(1:100)(Sigma–Aldrich) for 2 h at room temperature. Cell nuclei were labeled with cell permeable fluorescent DNA dye DraQ5 (Cell Signaling Technology). Cell images were captured with fluorescence microscopy (automatic microscope Olympus IX83; Olympus Optical Co., Ltd., Tokyo, Japan, and CellFamilyAnalySIS software) at 40× magnification.

### 4.11. Data Analysis

Data were presented as the mean value and standard error of the mean (SEM). Data were analyzed by means of GraphPad Prism 8 software (GraphPad Software, Inc., La Jolla, CA, USA). Statistical analysis was performed using one-way ANOVA with Tukey post hoc test or unpaired Student;s t-test; *p*< 0.05 was considered statistically significant. The IC50 value was calculated using a AAT BioquestQuest Graph™ IC50 Calculator (AAT Bioquest, Inc., Sunnyvale, CA, USA; https://www.aatbio.com/tools/ic50-calculator). Western blots were quantified densitometrically using NIH ImageJ software (Wayne Rasband, Bethesda, MD, USA) and are shown as % of control (the changes ≥20% were considered as significant). The data were normalized relative to β-actin.

## 5. Conclusions

In this study, we reported for the first time anti-proliferative and anti-migratory activity of 8-hydroxyquinaldic acid towards colon cancer HT-29 and LS-180 cells. It inhibited not only proliferation and mitochondrial activity, but also decreased DNA synthesis in both colorectal cell lines tested. On the molecular level, 8-hydroxyquinaldic acid induced changes in protein expression of cell cycle regulators (cyclin D1, cyclin E, CDK4, CDK6) and inhibitors of cyclin-dependent kinases (p21 Waf1/Cip1, p27 Kip1). Moreover, 8-hydroxyquinaldic acid inhibited migration of colon cancer HT-29 and LS-180 cells and increased the expression of β-catenin and E-cadherin. Importantly, anti-proliferative and anti-migratory concentrations of 8-hydroxyquinaldic acid were non-toxic in vitro and in vivo.

## List of Abbreviations

**ATCC**	American Type Culture Collection
**BrdU**	5′-bromo-2′-deoxyuridine
**CPT**	camptothecin
**CDK**	cyclin-dependent kinase
**CRC**	colorectal cancer
**DMEM**	Dulbecco’s modified Eagle’s medium
**ECACC**	European Collection of Authenticated Cell Cultures
**FBS**	fetal bovine serum
**FITC**	fluorescein isothiocyanate
**hpf**	hour postfertilization
**HUVEC**	Human Umbilical Vein Endothelial Cells
**LDH**	Lactate Dehydrogenase
**MAPK**	mitogen-activated protein kinase
**MTT**	3-(4,5-dimethylthiazol-2-yl)-2,5-diphenyltetrazolium bromide
**PBS**	phosphate buffered saline
**PI3K/Akt**	phosphoinositide 3-kinase/protein kinase B
**SDS**	sodium dodecyl sulphate
**TBS-T**	Tris-buffered saline–0.1% Tween 20
